# A Rare Case of Takotsubo Syndrome and Acute Coronary Syndrome of the Right Coronary Artery

**DOI:** 10.1155/2019/9128273

**Published:** 2019-06-09

**Authors:** Nicholas Mencer, Larry Todd Justice, William Black, Kayleigh Litton

**Affiliations:** ^1^Department of Interventional Cardiology Methodist Medical Center, Oak Ridge, Tennessee 37748, USA; ^2^Department of Interventional Cardiology, The University of Tennessee Knoxville, Knoxville, Tennessee 37996, USA

## Abstract

Takotsubo syndrome (TTS) is an increasingly recognized heart disease that was initially regarded as a benign condition, but since has proven to cause irreversible myocardial damage, resembling that of acute coronary syndrome (ACS). The etiology of TTS is still uncertain but may be associated with catecholamine elevations during times of emotional or physical stress. Catecholamines are also understood to have prothrombotic properties, which could lead to ACS. With these similarities, differentiating these two pathologies can be difficult, especially when TTS and ACS occur simultaneously.

## 1. Introduction

The pathogenesis of takotsubo syndrome (TTS) is not completely understood. Proposed mechanisms involve catecholamine excess during times of physical or emotional stress that lead to left ventricular myocardial dysfunction by direct catecholamine-associated toxicity and microvascular spasm [1, 2]. Although TTS may sometimes present as an ST elevation myocardial infarction (STEMI), angiography typically reveals no occlusive lesion within the coronary arteries. We present a case of a postmenopausal Caucasian female with TTS and coexisting critical stenosis of the right coronary artery (RCA).

## 2. Case Report

**Figure 1:**
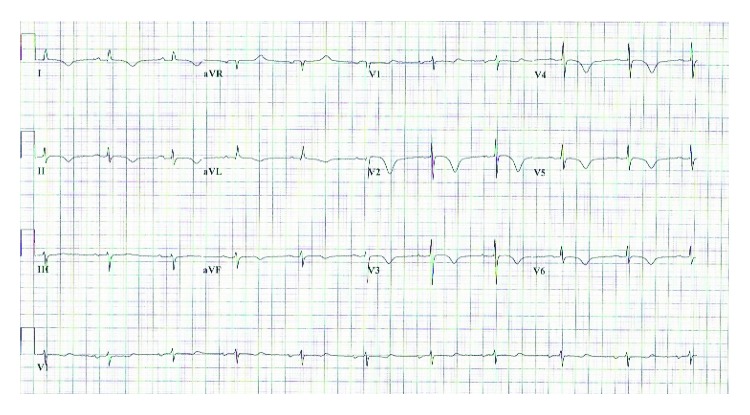
ECG on admission demonstrating diffuse T-wave inversions.

**Figure 2:**
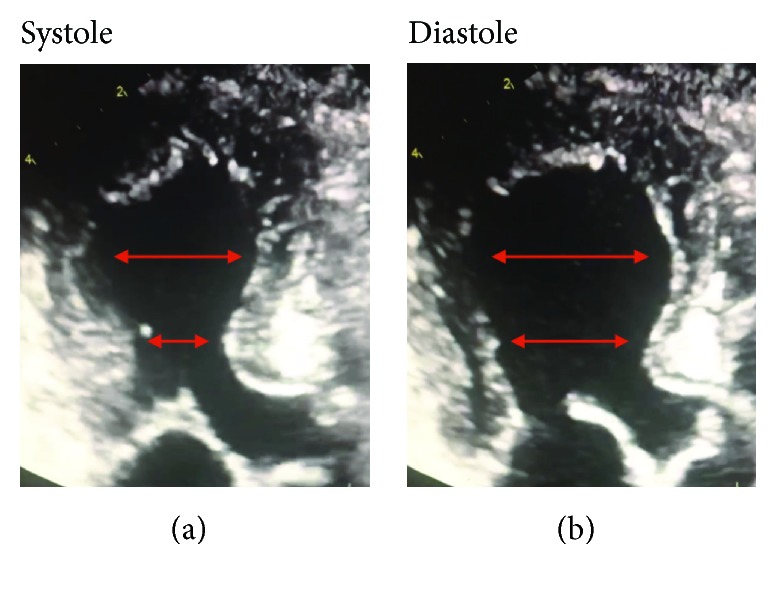
Apical three chamber echocardiogram demonstrating left ventricular apical ballooning and basal hyperkinesis consistent with takotsubo syndrome.

**Figure 3:**
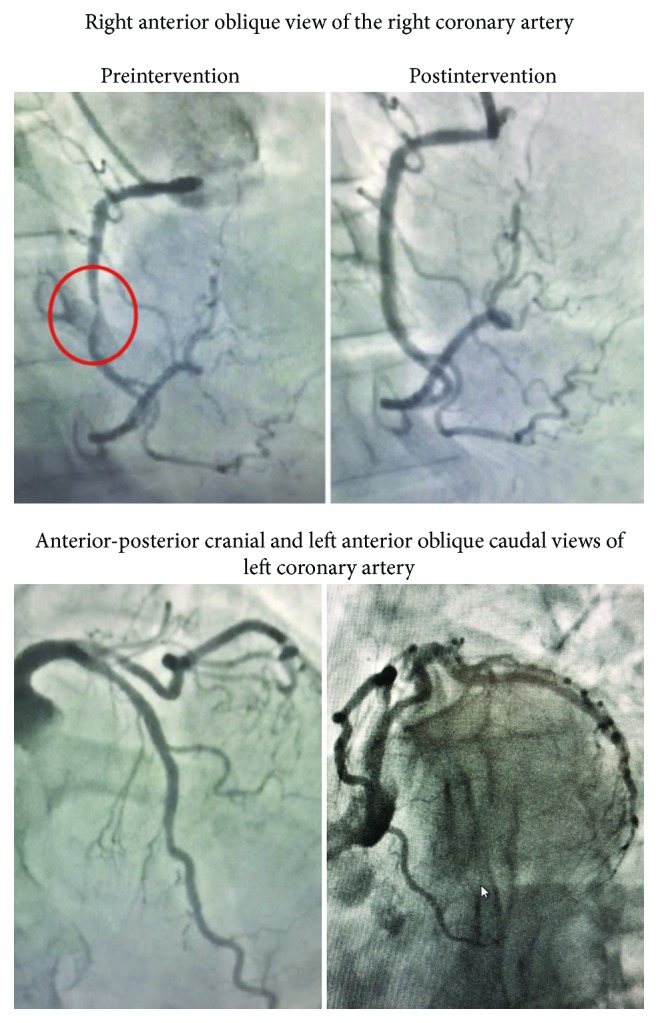
Angiographical images obtained during cardiac catheterization.

**Figure 4:**
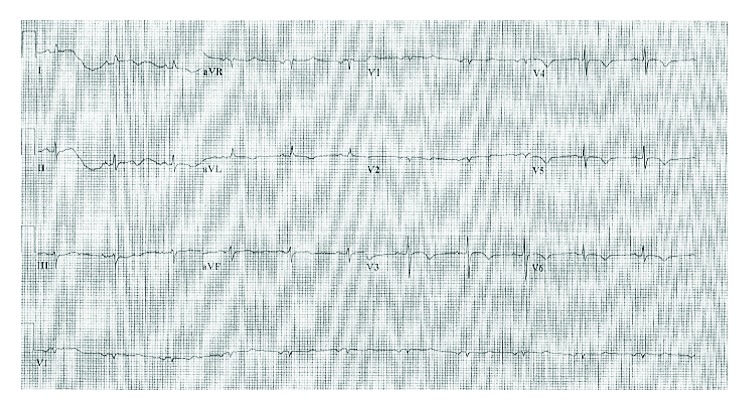
ECG post-PCI demonstrating interval improvement of inferior T-wave inversions and sustained T-wave abnormalities in the anterolateral leads.

A previously healthy 89-year-old female presented to the emergency department complaining of midsternal chest discomfort that radiated to her back. Her chest discomfort began the day prior to presentation, but she initially attributed it to indigestion and thus waited to seek medical attention. Her chest pain persisted however, which prompted her to seek evaluation in the emergency department. Upon arrival, she was given aspirin with resolution of her symptoms. Laboratory analysis revealed an elevated troponin I level of 0.319 and initial creatine kinase (CK) of 12.7. Brain natriuretic peptide level on presentation was not checked. Electrocardiogram (ECG) was notable for diffuse T-wave inversions demonstrating inferior, as well as anterolateral ischemia, and a prolonged QTc of 503 ms (Figure 1). Echocardiogram demonstrated moderate left ventricular dysfunction (ejection fraction of 35%-40%) with mid to distal anteroseptal, anterolateral, and apical akinesis (Figure 2). The patient was taken to the catheterization suite and underwent emergent left heart catheterization. Angiography revealed 90% stenosis in the mid right coronary artery (RCA) which was believed to be the culprit lesion. There were no significant obstructive lesions noted in the left anterior descending or left circumflex arteries. She underwent percutaneous coronary intervention (PCI) with successful stent placement to the RCA (Figure 3). She was then started on appropriate guideline-directed medical therapy and observed in the intensive care unit where her chest pain resolved. Repeat laboratory analysis revealed that CK had decreased to 6.4. ECG obtained following PCI revealed interval improvement of the inferior T-wave inversions, with sustained T-wave abnormalities in the anterolateral leads (Figure 4). Cardiac magnetic resonance imaging is not available at our facility and was not performed. The remainder of her hospitalization proceeded without incident, and she was discharged home in stable condition three days later.

**Figure 5:**
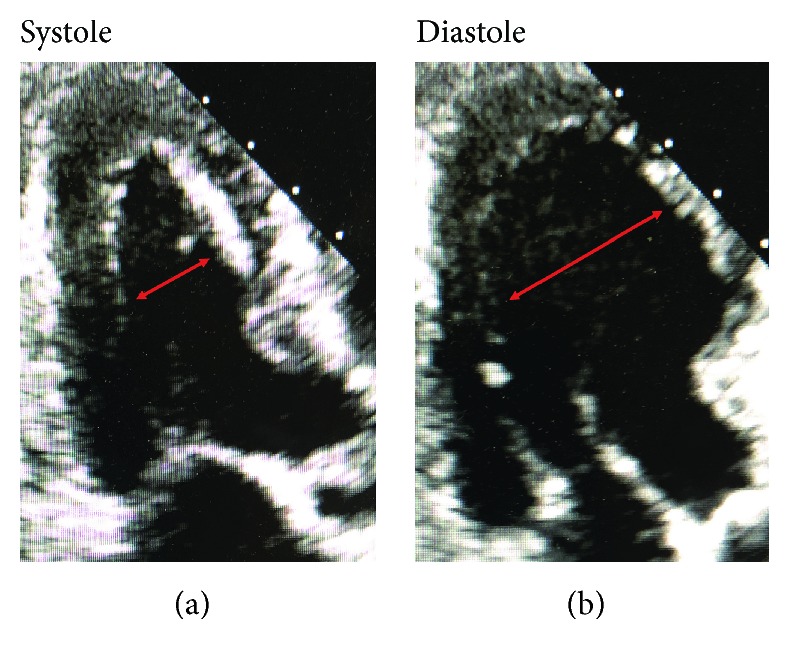
Apical three chamber echocardiogram obtained two weeks following revascularization of the RCA demonstrating no residual left ventricular dysfunction.

The patient was evaluated two weeks after discharge in an outpatient clinic and was feeling well. She again denied any stressors prior to the onset of pain but reported that the development of chest pain caused her a great deal of emotional distress. Repeat echocardiogram was obtained which revealed normal left ventricular systolic function (Figure 5).

## 3. Discussion

There have been numerous case reports of patients presenting with profound left ventricular dysfunction after suffering severe emotional or physical stress. This syndrome was first described in Japan in 1990 and is commonly referred to as transient apical ballooning syndrome or takotsubo syndrome [3–8]. Takotsubo syndrome is often associated with emotional triggers but has frequently been described in association with physical triggers. A large registry of patients with TTS found that physical triggers were more common than emotional triggers—women were more likely to have emotional triggers whereas men were more likely to have a physical trigger [9]. Although the diagnosis of TTS has historically been based on the presence of angiographically normal coronary arteries, there have been multiple reports of TTS occurring in the setting of physiological stress from acute coronary syndrome (ACS) [10]. The prevalence of TTS is estimated to be 2%-3% in patients who present with ACS and may be underestimated in individuals with coexisting coronary artery disease [11]. The recently updated International Takotsubo Diagnostic Criteria (InterTAK Diagnostic Criteria) specifically states that “significant coronary artery disease is not a contraindication in takotsubo syndrome” [1]. The exact pathophysiology of myocardial dysfunction due to sympathetic stimulation is unknown, but numerous mechanisms have been proposed. One potential mechanism is direct myocardial ischemia due to coronary arterial spasm—it has been shown that increased mental stress can cause vasoconstriction in patients without coronary artery disease [12]. Another potential mechanism is sympathetically mediated microvascular dysfunction resulting in abnormal coronary blood flow in the absence of obstructive disease [13]. A third potential mechanism is direct myocyte injury due to increased levels of circulating catecholamines which have been shown to cause contraction band necrosis. Similar injury patterns have been reported with catecholamine excess due to pheochromocytoma and subarachnoid hemorrhage [14, 15].

However, recent review suggests that although catecholamine excess may be linked to TTS, there is no direct causality [16].

This case poses two interesting questions: is the physiologic and emotional stress of chest pain enough to cause TTS? Can the catecholamine excess from TTS cause ACS? Catecholamines have been demonstrated to have prothrombotic properties in coronary arteries. Lin and Young demonstrated that by raising the plasma epinephrine concentration to approximately 27 nmol/L, the incidence of cyclic blood flow reductions in the coronary arteries of canines increased by 60% [17]. Catecholamine levels in takotsubo patients are 2-3 times higher than myocardial infarction patients 1-2 days after the onset of symptoms and 20 times higher than normal adults [18]. Additionally, the catecholamine levels in takotsubo patients remain elevated over myocardial infarction levels for 7-9 days [19]. With the known prothrombotic properties of catecholamines and the suspected rise in catecholamines associated with TTS, it is possible that TTS could have occurred first in our patient, leading to ACS of the RCA. This could have occurred either by thrombosis in our patient's native coronary artery or with the acute left ventricular (LV) systolic dysfunction associated TTS, an embolic event from a LV thrombus. However, extensive review of our patient's history, cardiac imaging, laboratory findings, and medical record failed to elucidate a possible cause for TTS other than her ACS.

Based on her description of symptoms and failure to identify another cause, we hypothesize that she initially developed ACS of the RCA, and the emotional distress from this event is likely the etiology of her TTS. Previous case series have reported that postischemic myocardial stunning has features typical of TTS and suggested that ACS may trigger TTS [20]. It can be difficult to distinguish between TTS and ACS as they have similar clinical presentations, and both can cause transient wall motion abnormalities [21]. Cardiac magnetic resonance imaging (CMR) can help distinguish TTS from myocardial infarction when there is diagnostic uncertainty, but unfortunately, we were unable to perform this in our patient [22].

## 4. Conclusion

The diagnosis of TTS has historically been a diagnosis of exclusion that required the presence of normal coronary arteries, but recent expert consensus has been updated to remove CAD as an exclusion criterion. Previous case reports have described cases of TTS coexisting with ACS as well as cases of ACS triggering TTS. This case contributes to the growing body of case reports that suggest that TTS and ACS can occur concomitantly.
